# Making “CASES” for AI in Medicine

**DOI:** 10.34133/bmef.0036

**Published:** 2024-01-29

**Authors:** Ge Wang

**Affiliations:** Biomedical Imaging Center, Center for Computational Innovations, Center for Biotechnology and Interdisciplinary Studies, Department of Biomedical Engineering, School of Engineering, Rensselaer Polytechnic Institute, Troy, NY, USA.

## Abstract

In this perspective, “CASES” are made for AI in medicine. The CASES mean Confidence, Adaptability, Stability, Explainability, and Security of AI systems. We underline that these CASES can be addressed not only individually but also synergistically on the large model platform and using cutting-edge diffusion-type models.

In recent years, artificial intelligence (AI) has been playing an important role in medicine and healthcare, revolutionizing many things from screening and diagnosis to therapy and prognosis [1]. This paradigm shift is largely due to deep artificial neural networks that analyze vast amounts of diverse data, recognize hidden patterns, and make predictions in a data-driven fashion. Despite its huge potential to go beyond human capabilities, the translation of AI into clinical practice still faces challenges. To address some key challenges conveniently, we propose the acronym “CASES” for Confidence, Adaptability, Stability, Explainability, and Security, discuss associated results, and envision future opportunities.

Confidence (or uncertainty): A key requirement for AI in clinical settings is to give confidence levels in its decisions. Medical professionals need to trust AI’s recommendations, which is often hindered by the inherent uncertainty in AI predictions. Uncertainty (aleatoric, epistemic, and numeric) arises due to various factors like data quality, algorithmic limitation, and biological and pathological complexity. While there are rigorous theory and effective methods in classic statistics to build confidence intervals, the gap is well recognized between classic statistics and contemporary AI especially deep learning [2]. To solve this problem, uncertainty quantification of AI systems is being actively researched to provide a confidence score alongside each deep prediction. Dropout [3] (randomly discarding neurons or breaking neural links during training) is the most popular technique to assess the uncertainty of a deep network output, which has been recently extended by us to Flipover [4] (randomly inverting the sign of some scaled neural outputs for adversarial defense). More sophisticated methods include conformal prediction [5] and recently published prediction-powered inference [6].

Adaptability (closely related to generalizability/transfer learning/domain adaptation): Adaptability refers to the ability of an AI system to perform accurately across related but different datasets. Often times, AI models trained on specific datasets may not generalize well, leading to decreased performance in other contexts [7]. This challenge can be tackled through transfer learning and domain adaptation, allowing AI models to avoid short-cut learning and adapt to new, unseen datasets without substantially compromising the performance [8]. To enhance the adaptability of AI systems, they should be trained using well-designed strategies, validated, and tested on diverse datasets that include various patient demographics, disease spectrums, and environmental factors [9].

Stability (robustness or reliability): Stability in AI refers to the robustness of its predictions when the input to a deep neural network is slightly changed by natural or adversarial noise [10]. Interestingly, adversarial instability is observed in not only deep image classification but also deep tomographic reconstruction [11]. In medicine, where patients’ lives are at stake, it is crucial that AI systems maintain reliable performance by consistently minimizing the first and second types of errors in presence of interferences. Along this direction, we developed an Analytic, Compressive, Iterative and Deep (ACID) approach to address the instability of deep image reconstruction networks [12]. Also, an independent empirical study reported that “*the existence of adversarial examples in classification tasks does not always carry over to NN-based solvers for inverse problems. Such reconstruction schemes may achieve state-of-the-art accuracy and can also, in certain cases, exhibit a similar degree of robustness as classical methods*.” [13] Furthermore, a latest theoretical approach was established with proven convergence and clear interpretability in the case of sparse data tomographic reconstruction [14].

Explainability (also referred to as interpretability): Explainability or interpretability is crucial in healthcare. Medical professionals are more likely to trust and adopt AI solutions that provide transparent and understandable decision-making processes. This means that AI models should not only be accurate but also provide insights about how they arrive at their conclusions. Techniques like feature scores and visualization tools can aid in making AI decisions more interpretable, as surveyed in [15]. However, it was recently noticed that not all interpretation tools are reliable; for example, see [16]. Several interesting schemes were proposed for principled interpretation of deep neural networks such as [17], but it remains open how to completely open the black box of deep learning.

Security (that is, patient privacy and confidentiality of other sensitive information): Security, particularly patient data confidentiality, is of paramount importance in hospitals and clinics. The integration of AI in medicine often involves the handling of sensitive patient data, which must be protected from unauthorized access and breaches. This requirement appears contradicting to the need for big data for deep learning such as in a healthcare metaverse [18] and has thus promoted development of secure computing and federated learning [19].

In the above discussions, the CASES are made individually but each of the five issues is intrinsically entangled with the other issues. Hence, it would be optimal to make the CASES synergistically, as shown in Fig. 1. Fortunately, the emerging large language models and multimodal large models in the medical domain seem the ideal platform to address these problems together [20]. These large models, capable of processing and correlating information from diverse data types (like images, text, and genomic data), hold great potential for personalized medicine. In our own study on the first Large Image-Text model to handle volumetric chest computed tomography images, we found that the AI performance on multiple tasks as a whole is significantly better than the separated counterparts on these tasks [21]. Another exciting development in the deep learning field is generative AI via diffusion models that generally outperform the famous generative adversarial networks [22]. While federated learning only shares network parameters, we believe that sharing could be enabled at the raw data level using an enhanced diffusion model embedded with a security safeguard mechanism [23]. Since the diffusion models have a solid theoretical bases (stochastic differential equation, thermodynamics, and electrostatics), their elegant properties facilitate making the CASES effectively but at a more computational cost. To overcome this shortcoming, active research is going on to accelerate the sampling speed of the diffusion models such as using consistency model and Poisson flow generative models [24].

Typically, DL stands for deep learning. Now, it is also fine to consider D for diffusion-type models and L for large hybrid models. These and other frontiers are rapidly evolving toward a brighter future of medicine and healthcare. Specifically, the integration of AI into medicine after effectively addressing the CASES issues presents an unprecedented opportunity to facilitate clinical throughput, improve patient outcomes, and eliminate healthcare disparities.

**Fig. 1.**
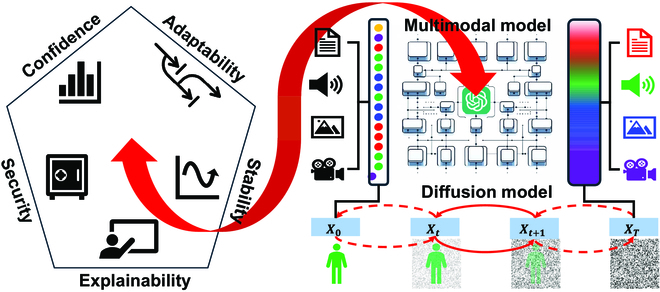
Confidence, Adaptability, Stability, Explainability, and Security are important for AI in medicine and can be improved using emerging diffusion-type modes and multimodal large models.
